# Hepatic arterial infusion chemotherapy versus transarterial chemoembolization for unresectable hepatocellular carcinoma: A systematic review with meta-analysis

**DOI:** 10.3389/fbioe.2022.1010824

**Published:** 2022-09-27

**Authors:** Tengfei Si, Zhenlin Huang, Shirin Elizabeth Khorsandi, Yun Ma, Nigel Heaton

**Affiliations:** ^1^ Department of Inflammation Biology, Faculty of Life Sciences & Medicine, Institute of Liver Studies, King’s College Hospital, King’s College London, Denmark Hill, London, United Kingdom; ^2^ The MOE Key Laboratory for Standardization of Chinese Medicine, Shanghai University of Traditional Chinese Medicine, Shanghai, China; ^3^ Transplant Services, King’s College Hospital, Denmark Hill, London, United Kingdom; ^4^ The Roger Williams Institute of Hepatology, Foundation for Liver Research, London, United Kingdom

**Keywords:** hepatocellular carcinoma, infusion, transarterial chemoembolization, metaanalysis, comparison

## Abstract

**Background:** Interest has revived in the use of hepatic arterial infusion chemotherapy (HAIC) for intermediate-advanced hepatocellular carcinoma (HCC) while transarterial chemoembolization (TACE) has been a longstanding loco-regional therapy.

**Aim:** We conducted a systematic review and meta-analysis of patients with unresectable HCC treated with HAIC or TACE to look for differences in survival, adverse events, mortality and downstaging.

**Methods:** All studies published before 29 July 2022 were identified by searching PubMed, Embase, Web of Science and Cochrane Library databases for patients with unresectable HCC and received HAIC or TACE as initial treatment. Data extracted from studies was statistically analysed using RevMan5.3 software.

**Results:** A total of one randomized controlled trial (RCT) and 7 cohort studies (5 retrospective, 2 prospective) including 1,060 (TACE group: 534, HAIC group: 526) patients were screened. Compared with the TACE group, patients who received HAIC as initial therapy had better overall survival (OS) (HR = 0.53, 95%CI [0.40, 0.69]) and progression-free survival (PFS) (HR = 0.54, 95%CI [0.40, 0.72]). Further subgroup analysis revealed that HAIC showed priority over TACE on prognosis outcome regardless of tumour stage, especially in patients with advanced portal vein tumour thrombus (PVTT). Utilization of port system will not boost the efficacy of HAIC whereas using a replaced-microcatheter for each procedure could better reduce the progressive disease (PD) rate (RR = 0.55, 95%CI [0.40, 0.76]). The pooled RR favoured the HAIC group with regard to partial response (PR) (RR = 2.87, 95%CI [2.18, 3.78]) and this was validated by both GRADE summary and trial sequential analysis. The rate of resection after treatment was higher in the HAIC group (RR = 2.37, 95%CI [1.54, 3.66]), whilst no difference was found with procedure-related mortality (RR = 0.56, 95%CI [0.13, 2.38]) between two groups. Compared with the traditional chemotherapy regimen (fluorouracil/leucovorin/oxaliplatin) FOLFOX-HAIC appears to be better in improving the treatment efficacy.

**Conclusion:** Patients with unresectable HCC could potentially benefit more from HAIC rather than standard TACE treatment. A re-evaluation of HAIC as a treatment option in intermediate and advanced HCC is warranted.

## Introduction

Hepatocellular carcinoma (HCC) is one of the most common gastrointestinal malignancies. Early HCC are asymptomatic and many patients present late with large lesion. For patients with Barcelona clinic liver cancer (BCLC) stage B HCC, transarterial chemoembolization (TACE) is the first line loco-regional treatment. However, for those with portal vein tumour thrombus (PVTT) or tumour size over 10 cm in diameter, TACE may be contraindicated or considered to be of limited benefit.

A small number of studies have shown that for large unresectable HCC or HCC refractory to TACE or sorafenib, hepatic arterial infusion chemotherapy (HAIC) may provide longer progression-free survival (PFS) and achieve a higher rate of disease control (Shao et al., 2013; Obi et al., 2015). It has been used in advanced HCC with reports of similar or better treatment response than sorafenib monotherapy (Choi et al., 2018; Kodama et al., 2018; Moriya et al., 2018; Ueshima et al., 2020; Ahn et al., 2021). However, there is a lack of randomized clinical trials or prospective studies of using HAIC as first-line treatment in HCC. Management guidelines of the American Association for the Study of Liver Diseases (AASLD), the European Society of Liver Diseases (EASL) and BCLC have not considered its clinical application for HCC (Llovet et al., 2004; European Association for the Study of the Liver, 2018; Marrero et al., 2018) whereas in China, South Korea and Japan, HAIC has been commonly used for intermediate-advanced HCC, especially in those patients with a poor response to TACE or sorafenib (Kudo et al., 2014; Korean Liver Cancer and National Cancer, 2018; Shiina et al., 2020).

To date, only a small number of studies have evaluated the efficacy of HAIC in treating HCC compared with conventional TACE. These studies support HAIC as potential treatment for advanced HCC whereas for intermediate stage HCC, data is lacking (Choi et al., 2018; Kodama et al., 2018; Moriya et al., 2018; Ueshima et al., 2020; Ahn et al., 2021). Therefore, we combined all available studies to systematically assess the effectiveness and safety of HAIC in comparison to TACE in treating intermediate-advanced HCC.

## Materials and methods

The study was performed according to a pre-registered protocol at the International Prospective Register of Systematic Reviews (PROSPERO) (Available from: https://www.crd.york.ac.uk/prospero/display_record.php?ID=CRD42021248823). This systematic review and meta-analysis was reported following the Preferred Reporting Items for Systematic Reviews and Meta Analyses statement suggested by the Cochrane handbook (Page et al., 2021).

### Searching strategy

Databases including PubMed, Embase, Cochrane Library and Web of Science were searched to collect all available studies where HAIC and TACE were utilised to treat unresectable HCC. The primary search strategy was based on medical subject headings terms (MeSH), combined with free text words. The following key words were used: “Hepatic arterial infusion chemotherapy,” “HAIC,” “transarterial chemoembolization,” “TACE,” “hepatocellular carcinoma,” “HCC,” “Liver cance.r” The searching cut-off date was 29 July 2022. We also checked the reference lists of all identified studies for additional eligible data.

### Inclusion and exclusion criteria

Unresectable HCCs in this study refers to large HCC considered to have insufficient remnant liver volume after potential resection, advanced HCC with main portal invasion, diffuse bilobar involvement and/or refractory to radical treatment (liver transplant, hepatectomy or ablation). The detailed inclusion criteria for patient enrolment in each study is shown in Supplementary Table S3. Any studies comparing the clinical outcomes of patients with unresectable HCC who had HAIC or TACE as their initial treatment were included. The language of published literatures was limited to English only. Patients with metastatic liver secondaries (non-HCC), receiving adjuvant therapy besides TACE/HAIC or with extrahepatic primary malignancy were excluded. For repeated publications or overlapping cases, the publication which had more comprehensive information was included. Abstracts, letters, editorials, expert opinions and reviews were excluded to ensure only original data were used.

### Data extraction and study assessment

Data extraction from each study was performed by two authors independently (TFS&ZLH). Patients’ basic characteristics, overall survival (OS), progression-free survival (PFS), details of interventions, tumour response after treatment including complete response (CR)/partial response (PR)/stable disease (SD)/Progressive Disease (PD), adverse events and intervention related mortality data were extracted from each study using a pre-designed data extraction form. For missing information, an attempt was made to contact authors of original articles. During the process of data extraction, any disagreement was resolved by discussion or with a third reviewer (YM) if necessary.

The study quality assessment/risk of bias analysis was conducted by two reviewers independently (TFS&ZLH). The Newcastle-Ottawa Scale (NOS) was used to evaluate the quality of observational studies, a maximum of one star could be aliquoted for each numbered item within the Selection and Outcome categories, while a maximum of two stars was given for Comparability (Zeng et al., 2015). Each study was awarded 0–9 stars, with 0–3, 4–6 and 7–9 considered as low, moderate and high quality respectively for NOS scale. The risk of bias graph and summary suggested by Cochrane Handbook was used for randomised controlled studies (RCT). The Grading Recommendations Assessment, Development and Evaluation (GRADE) approach was used to assess the certainty of evidence for meta results (Guyatt et al., 2008). Guideline Development Tool was accessed from https://www.gradepro.org to create the Summary of Findings table. During the process of quality assessment, disagreement was resolved by discussion or with a third reviewer if necessary (YM).

### Statistical analysis

Quantitative analysis was performed using Review Manager (version 5.3.5 for Windows). We used hazard ratio (HR) to evaluate patients’ OS and PFS. The overall pooled HR and 95% confidence interval (CI) were calculated with the inverse variance method described by Tierney et al. (2007). Dichotomous variables were tested by risk ratio (RR) with a 95% CI. All data were calculated with a random effect model. A value of *p* < 0.05 was considered as statistically significant. 95% CI for the pooled HR and RR did not overlap 1 was equivalent to a *p*-value less than 0.05. Heterogeneity between studies was measured by chi-squared test and I^2^, comparisons with a *p* value less than 0.1 were defined as statistically heterogeneous. A brief guide to interpretation of the I^2^ statics is as follows: 50%–90% may represent substantial heterogeneity, 75%–100% as considerable heterogeneity. For those results with statistical heterogeneity, a subgroup or sensitivity analysis was performed to identify the source of heterogeneity when appropriate. Publication bias was assessed through both visual inspection of funnel plots or the rank correlation test of Begg and the regression asymmetry test of Egger when necessary (if *n* > 10). Trial sequential analysis (TSA) was conducted for outcomes with high/moderate quality after GRADE assessment. The significance level was set at 5% with a power of 80% and a relative risk reduction (RRR) of 25%. Model variance-based heterogeneity correction was applied using Trial Sequential Analysis v.0.9.5.10 beta software (Copenhagen Trial Unit, Centre for Clinical Intervention Research https://www.ctu.dk/tsa) (Wetterslev et al., 2017).

## Results

### Characteristics of the included studies

**FIGURE 1:**
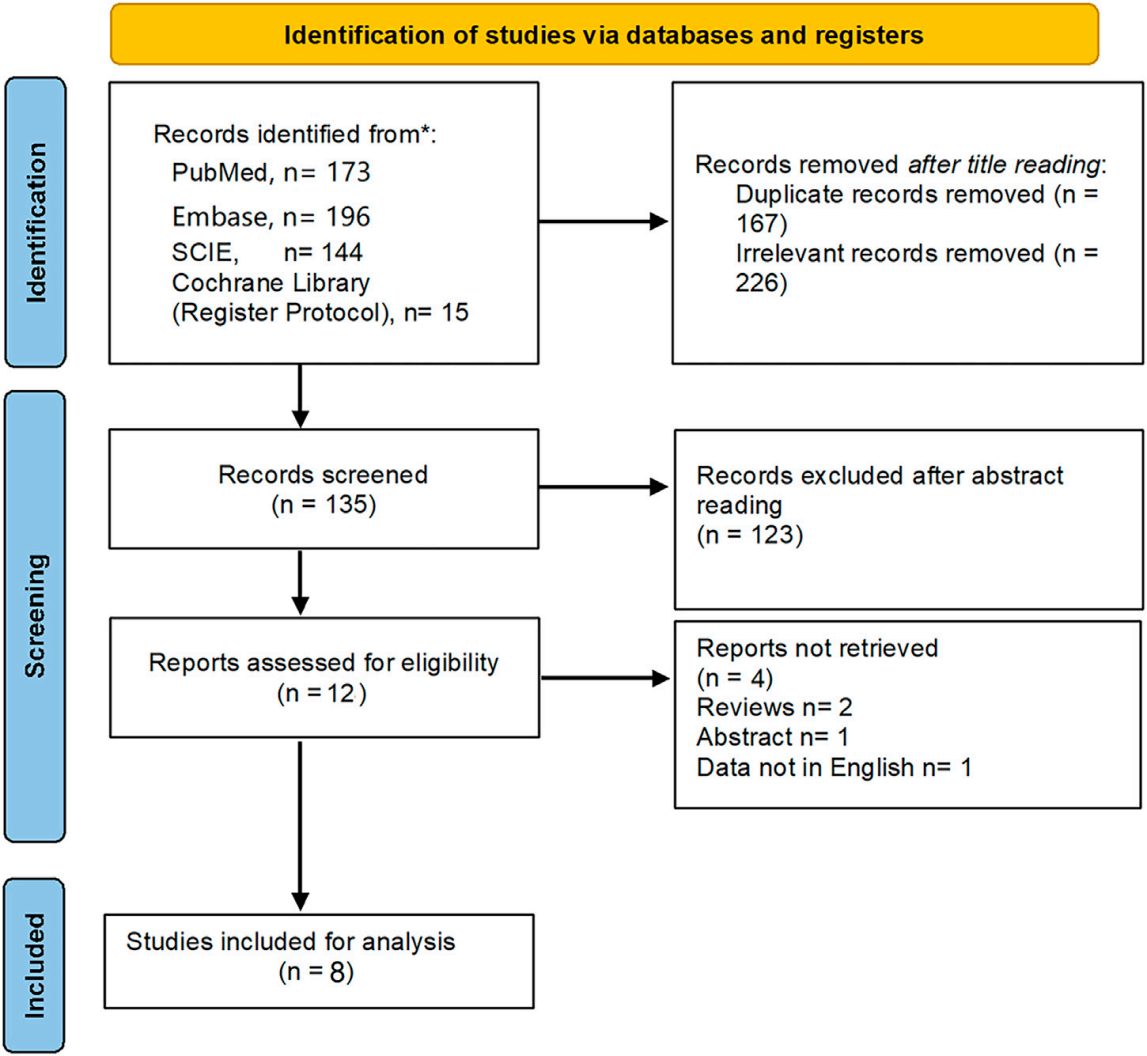
The flowchart of study selection.

According to the searching strategy (Supplementary Table S1), 528 titles were identified from four databases. Finally, one RCT and 7 cohort studies met with inclusion criteria after full-text reviewing were included (Sumie et al., 2003; Kim et al., 2010; He et al., 2017; Hu et al., 2020; An et al., 2021; Li et al., 2021; Chen et al., 2022; Li et al., 2022). Data of 1,060 patients (HAIC, *n* = 526; TACE, *n* = 534) from Japan, South Korea and China was extracted for quantitative analysis (Sumie et al., 2003; Kim et al., 2010; He et al., 2017; Hu et al., 2020; An et al., 2021; Li et al., 2021; Chen et al., 2022; Li et al., 2022). Figure 1 shows the detailed study selection process. The baseline characteristics and intervention details of included studies are shown in Tables 1, 2. The comparisons between the HAIC and TACE groups included primary outcomes: patients’ survival and treatment response, secondary outcomes: mortality, downstaging and adverse event after interventions.

**TABLE 1:** Baseline characteristics of included studies in the meta-analysis.

Study	Study design	Group	Patients	Gender (M/F)	Age	HBV (Y/N)	Child pugh (A/B/C)	BCLC (A/B/C)	AFP (ng/ml)	Tumour size (cm)
Sumie et al. (2003)	Retrospective	HAIC	16	12/4	66.5 (46–76)	1/15	7/9/0	n/a	81 (9–47,000)	≤5, *n* = 11
										>5, *n* = 5
		TACE	21	16/5	67 (36–80)	2/19	13/8/0	n/a	290 (1.7–120,000)	≤5, *n* = 14
										>5, *n* = 7
Kim et al. (2010)	Prospective	HAIC	36	n/a	53 ± 10.9	30/6	33/3/0	0/12/19	1,561 (3.2–155800)	12 ± 4 (5.5–24.8)
		TACE	31	n/a	55 ± 9.2	26/5	20/11/0	0/5/31	1750 (3.12–195000)	13.8 ± 4 (10–26)
He et al. (2017)	Prospective	HAIC	38	30/8	≤60, *n* = 27	36/2	38/0/0	15/23/0	≤400, *n* = 12	<10, *n* = 12
					>60, *n* = 11				>400, *n* = 26	≥10, *n* = 26
		TACE	41	37/4	≤60, *n* = 27	36/5	41/0/0	11/30/0	≤400, *n* = 15	<10, *n* = 12
					>60, *n* = 14				>400, *n* = 26	≥10, *n* = 29
Hu et al. (2020)	Retrospective	HAIC	22	21/1	52.5 (43.5–59.0)	21/1	20/2/0	0/0/22	2019.5 (235.0–5,821.5)	7.7 (4.85–12.1)
		TACE	24	20/4	54 (49.0–62.0)	23/1	21/3/0	0/0/24	3,774.5 (151.9–15,032)	8.2 (7.05–13.5)
Li et al. (2021)	Retrospective	HAIC	101	89/12	50 (18–75)	90/11	n/a	0/0/101	≤400, *n* = 36	≤10, *n* = 39
									>400, *n* = 65	>10, *n* = 62
		TACE	131	121/10	52 (24–75)	114/17	n/a	0/0/131	≤400, *n* = 47	≤10, *n* = 83
									>400, *n* = 84	>10, *n* = 48
Chao et al. (2021)	Retrospective	HAIC	92	83/9	50.2 ± 11.3	73/19	92/0/0	0/0/92	<400, *n* = 27	10.8 ± 3.4
									≥400, *n* = 65	
		TACE	68	61/7	51.1 ± 13.1	65/3	68/0/0	0/0/68	<400, *n* = 25	11.0 ± 3.4
									≥400, *n* = 43	
Li et al. (2022)	RCT	HAIC	159	135/24	53 (44–63)	140/19	159/0/0	60/99/0	≤400, *n* = 83	≤10, *n* = 84
									>400, *n* = 76	>10, *n* = 75
		TACE	156	141/15	54 (43–62)	141/15	156/0/0	54/102/0	≤400, *n* = 75	≤10, *n* = 84
									>400, *n* = 81	>10, *n* = 72
Chen et al. (2022)	Retrospective	HAIC	62	54/8	53.2 ± 10.3	59/3	54/8/0	0/32/30	≤400, *n* = 23	≤10, *n* = 27
									>400, *n* = 39	>10, *n* = 35
		TACE	62	46/16	53.5 ± 12.7	59/3	55/7/0	0/37/25	≤400, *n* = 22	≤10, *n* = 27
									>400, *n* = 40	>10, *n* = 35

n/a, not available; AFP, alpha-fetoprotein; BCLC, barcelona clinic liver cancer; HAIC, hepatic arterial infusion chemotherapy; HBV, hepatitis B virus; RCT, Randomized controlled trial. TACE, trans-arterial chemoembolization.

**TABLE 2:** Intervention details of included studies.

Study	Group	Drugs and dosage	Courses	Interval	Termination
Sumie et al. (2003)	HAIC	Cisplatin (10 mg/person) for 1 h on days 1–5 followed by 5-fluorouracil (250 mg/person) for 5 h on days1–5	n/a	3 weeks	n/a
	TACE	Epirubicin (20–30 mg/person) and Lipiodol (2–4 ml)	n/a	3–4 weeks	n/a
Kim et al. (2010)	HAIC	5-fluorouracil (5-FU; 500 mg/m^2^) for 5 h on days 1–3 and cisplatin (60 mg/m^2^) for 2 h on day 2	3.4 ± 2.3 (1–11)	4 weeks	Until disease progressed or unacceptable toxicity was evident or withdraw consent
	TACE	Doxorubicin (10–60 mg) in a mixture of 5–10 ml of lipiodol and was partly accompanied by embolization using gelfoam in selected cases	1.7 ± 1.4 (1–8)	4–8 weeks	
He et al. (2017)	HAIC	Oxaliplatin, 85 mg/m^2^ intra-arterial infusion on day 1; Leucovorin, 400 mg/m^2^ intra-arterial infusion on day 1; and 5-FU, 400 mg/m^2^ bolus infusion on day 1 and 2,400 mg/m^2^ continuous infusion over 46 h	3.8 ± 1.5 (1–6)	3 weeks	Both were discontinued when disease progression or intolerable AEs occurred, or patient was eligible for another treatment (surgical resection) or withdrew consent or no recovery occurred after a 30-day delay
	TACE	50 mg of epirubicin +50 mg of lobaplatin and 6 mg of mitomycin C mixed with 10 ml of lipiodol, embolization was performed with the injection of polyvinyl alcohol particles that were 300–500 μm in diameter	1.7 ± 0.8 (1–3)	6 weeks	
Hu et al. (2019)	HAIC	Oxaliplatin (35–40 mg/m^2^) for 2 h followed by 5-FU (600–800 mg/m^2^ for 22 h) on days 1–3, 200 mg/m^2^ of leucovorin calcium for 2 h from the beginning of the 5-FU infusion	5 (2–9)	4 weeks	Patients received full 6 courses or severe liver function damage or with disease progression
	TACE	40–60 mg of epirubicin and 5–15 ml of lipiodol, embolization was performed with the injection of 150–350 µm/350–550 µm of gelatine sponge particle or 100–300 µm/300–500 µm mebospheres	1 (1–3)	4–6 weeks	No residual tumour or with contraindications for TACE
Li et al. (2021)	HAIC	Oxaliplatin 130 mg/m^2^ by intra-arterial over 2–4 h; leucovorin 200 mg/m^2^ infusion for 2 h, fluorouracil 400 mg/m^2^ bolus on day 1; 2,400 mg/m^2^ continuous i.a infusion over 46 h	4.2 (1–9)	3 weeks	Intrahepatic lesions progressed or toxicity became unacceptable
	TACE	60 mg of epirubicin and 5–20 ml of lipiodol, embolization was performed with the injection of gelatin sponge particles or 300–500 μm diameter polyvinyl alcohol particles	2.4 (1–12)	4 weeks	Doctors and the patient changed the subsequent therapy according to the follow-up results
Chao et al. (2021)	HAIC	Oxaliplatin (130 mg/m^2^ infusion for 3 h on day 1) + leucovorin (200 mg/m^2^ for 3–5 h on day 1) + Fluorouracil (400 mg/m^2^ in bolus, and then 2,400 mg/m^2^ continuous infusion 46 h)	4 (2–8)	3 weeks	Intrahepatic lesions progressed or toxicity became unacceptable
	TACE	10–20 ml lipiodol +30–50 mg lobaplatin +20–40-mg epirubicin, gel foam mixed with contrast medium was injected if necessary	2 (1–4)	4–6 weeks	n/a
Li et al. (2022)	HAIC	Oxaliplatin (130 mg/m^2^ from hour 0–2 on day 1) + leucovorin (400 mg/m^2^ from hour 2–3 on day 1) + fluorouracil (400 mg/m^2^ bolus at hour 3 on day 1 and 2,400 mg/m2 over 24 h)	3.6 (1.7)	3 weeks	tumour progression, the disappearance of any arterial enhancement in all intrahepatic lesions, intolerable toxicity, study treatment delays of more than 30 days, technical difficulty, the need for another anticancer treatment (such as surgery) at the physician’s discretion or at the patient’s request
	TACE	50 mg of epirubicin +50 mg of lobaplatin mixed with lipiodol + polyvinyl alcohol particles	2 (1.4)	6 weeks	
Chen et al. (2022)	HAIC	Oxaliplatin (100 mg/m^2^, continuous infusion for 4 h) + raltitrexed (3 mg/m^2^, continuous infusion for 1 h)	3.5 (2–8)	3 weeks	the disease progressed, the toxicity levels could not be tolerated, or the patient refused to continue treatment
	TACE	5–20 ml of lipiodol + Epirubicin with gelatin sponge particles (150–350, 350–560, and 560–750 μm)	2.4 (1–8)	4–6 weeks	

HAIC, hepatic arterial infusion chemotherapy; TACE, trans-arterial chemoembolization.

### Study assessment

7 cohort studies were rated with 5 stars or more, of which 2 studies were awarded 5-6 stars in accordance with median quality, while other five studies were awarded with 7-9 stars in accordance with high quality (Supplementary Table S2). As to the one RCT, the risk of bias details was shown in Supplementary Figure S1.

### Primary outcomes

#### Patients’ survival

**FIGURE 2:**
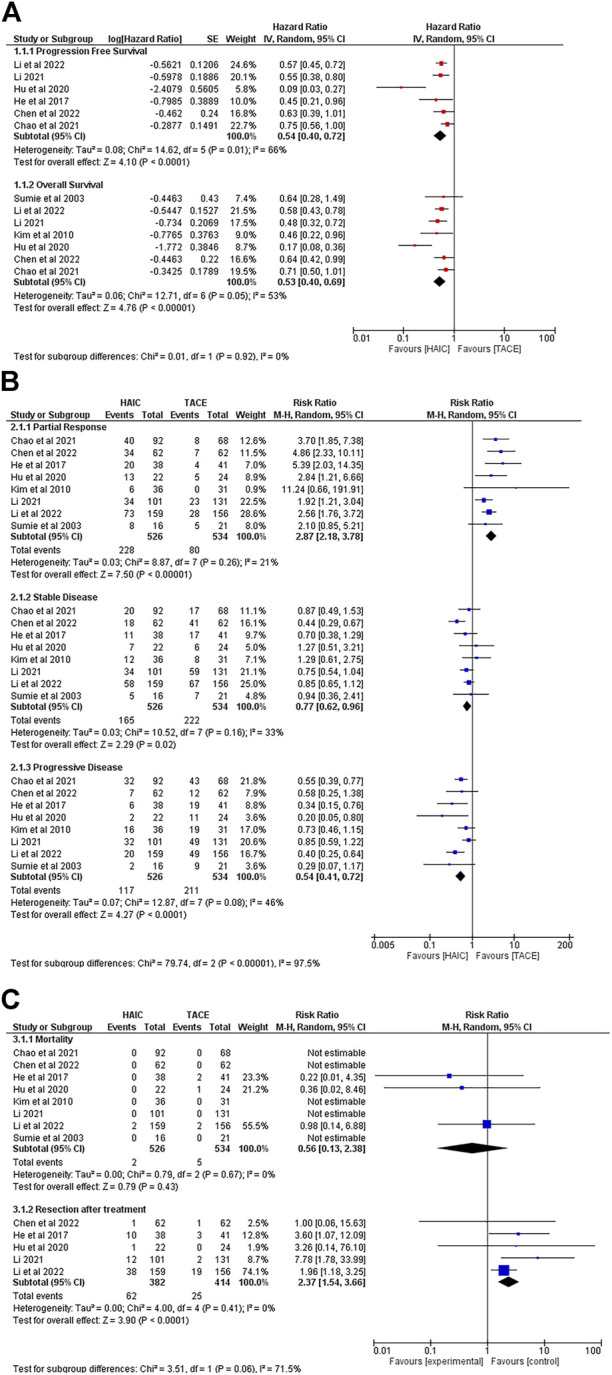
Comparisons of outcomes between the HAIC group and the TACE group. **(A)**. Forest plots of survival; **(B)** Forest plots of tumour response after treatment; **(C)** Forest plots of mortality and the rate of resection after interventions.

Six studies reported patients’ PFS (Sumie et al., 2003; Kim et al., 2010; Hu et al., 2020; An et al., 2021; Li et al., 2021; Li et al., 2022; Sumie et al., 2003; Kim et al., 2010; Hu et al., 2020; An et al., 2021; Li et al., 2021; Li et al., 2022; He et al., 2017; Li et al., 2021; Chen et al., 2022; Li et al., 2022) and 7 studies provided the time-to-event data regarding patients’ OS (Sumie et al., 2003; Kim et al., 2010; Hu et al., 2020; An et al., 2021; Li et al., 2021; Li et al., 2022; Sumie et al., 2003; Kim et al., 2010; Hu et al., 2020; An et al., 2021; Li et al., 2021; Li et al., 2022). Figure 2A presents the pooled HRs for PFS and OS. It showed that patients in the HAIC group had better OS (HR = 0.53, 95%CI [0.40, 0.69]) and PFS (HR = 0.54, 95%CI [0.40, 0.72]) compared to the TACE group. Significant heterogeneity was observed among the above comparison (*p* = 0.05, I^2^ = 53%; *p* = 0.01, I^2^ = 66%)

#### Treatment response

All 8 studies reported data regarding objective response rate (ORR). Of 1,060 patients, only two patients from the HAIC group reported a CR. The data showed no difference between the HAIC group and the TACE group in the rate of CR (RR = 3.88, 95%CI [0.41, 36.36], *p* = 0.23) (Supplementary Figure 2). With regards to PR, the pooled RR favoured the HAIC treated patients (RR = 2.87, 95%CI [2.18, 3.78]). Patients from the TACE group had better SD rate (RR = 0.77, 95%CI [0.62, 0.96]) whereas a higher risk of experiencing PD after treatment (RR = 0.54, 95%CI [0.41, 0.72]) (Figure 2B).

### Secondary outcomes

#### Intervention related mortality and sequent resection after treatment

Figure 2 summarizes the mortality data and the rate of subsequent surgical resection after interventions. Although no significant difference was observed in mortality between the HAIC group and TACE groups (RR = 0.56, 95%CI [0.13, 2.38]), 5 deaths occurred in the TACE group, and 2 in the HAIC group. During follow-up, more patients in the HAIC group achieved the goal of downstaging to subsequent surgical resection (RR = 2.37, 95%CI [1.54, 3.66]) (Figure 2C).

#### Adverse events after interventions

Six studies provided detailed information about severe adverse events after treatment. According to Common Terminology Criteria Adverse Event (v3&v4) (Trotti et al., 2003), only records for Grade III-IV events were collected. The pooled RRs showed that patients from the TACE group had a higher risk of experiencing severe fever (RR = 0.19, 95% CI [0.06, 0.64]), hyperbilirubinemia (RR = 0.23, 95%CI [0.07, 0.75]) and significantly elevated ALT level (RR = 0.28, 95% CI [0.12, 0.64]). No difference was found between the two groups in other adverse events such as leukopenia (RR = 2.37, 95%CI [0.75, 7.47]), diarrhoea (RR = 3.99, 95%CI [0.86, 18.56]), neutropenia (RR = 1.77, 95%CI [0.52, 6.00]), anemia (RR = 0.63, 95%CI [0.13, 3.12]) or thrombocytopenia (RR = 1.35, 95%CI [0.60, 3.02]) (Supplementary Figure S3).

### Subgroup and sensitivity analysis

Through the whole meta-analysis, the comparisons of prognosis between two groups were found with statistical heterogeneity. Therefore, subgroup and sensitivity analysis were performed to identity heterogeneity source. According to patients’ vascular invasion degree, studies were further divided into three subgroups: (Group A) all patients with Vp3 or Vp4 PVTT (Hu et al., 2020), (Group B) a small portion of patients with Vp3-Vp4 PVTT (Kim et al., 2010; Chao et al., 2021; Li et al., 2021) and (Group C) no patients with Vp3-Vp4 PVTT (Sumie et al., 2003; He et al., 2017; Li et al., 2022). All three subgroups showed that the HAIC group had better PFS and OS compared to the TACE group, and no statistical heterogeneity was found (Supplementary Table S4). In the sensitivity analysis, the pooled HRs and 95% CIs for OS and PFS after excluding Hu et al.’s study were significantly different from others. The study by Hu et al., which only included patients with Vp3 or Vp4 PVTT seemed to be the main source of heterogeneity in comparisons of survival (Supplementary Figure S4).

Differences in the degree of tumour progression and HAIC regimen or methods are compounding factors affecting patients’ prognosis. To avoid the influence of the above factors on the comparisons between the HAIC and TACE group, patients were divided according to BCLC stage and HAIC procedure. The results showed that:

1) In both BCLC stage A-B and stage C, the HAIC group showed better OS (HR = 0.58, 95%CI [0.43, 0.78]; HR = 0.42, 95%CI [0.22, 0.81]) and PFS (HR = 0.56, 95%CI [0.45, 0.70]; HR = 0.42, 95%CI [0.21, 0.86]), higher PR (RR = 3.23, 95%CI [1.63, 6.38]; RR = 2.51, 95%CI [1.65, 3.81]), lower PD (RR = 0.38, 95%CI [0.26, 0.58]; RR = 0.6, 95%CI [0.36, 0.98]) and higher sequent resection rate (RR = 2.15, 95%CI [1.35, 3.42]) compared to the TACE group (Table 3).
2) Whether using a port system for the chemotherapy treatment or not did not change the efficacy of HAIC, and HAIC still showed priority over TACE in terms of OS, PFS and PR rate. By using a replaced-microcatheter for each procedure rather than a fixed port system, HAIC appeared to have a lower risk of PD (RR = 0.55, 95%CI [0.40, 0.76]) and an increase in the subsequent resection rate (RR = 2.68, 95%CI [1.41, 5.08]) (Table 3).
3) HAIC using FOLFOX appears to be more effective than the combination of cisplatin plus fluorouracil in PR (RR = 2.67, 95%CI [2.00, 3.57]) and PD (RR = 0.50, 95%CI [0.34, 0.74]) when compared to the TACE group (Table 3).

**TABLE 3:** Subgroup analysis of outcomes.

Outcome/subgroup			OS	PFS	Treatment response			Mortality	Resection
					*PR*	*PD*	*SD*		
BCLC Stage subgroup analysis	BCLC A-B^a^	HR/RR	0.58	0.56	3.23	0.38	0.82	0.63	2.10
		95%CI	[0.43, 0.78]	[0.45, 0.70]	[1.63, 6.38]	[0.26, 0.58]	[0.64, 1.06]	[0.12, 3.21]	[1.33, 3.32]
		*p* value	***0.0004***	***<0.00001***	***0.0008***	***<0.00001***	0.13	0.57	***0.002***
		I^2^	n/a	0%	49%	0%	0%	0%	0%
	BCLC C^b^	HR/RR	0.42	0.42	2.51	0.6	0.81	0.36	6.66
		95%CI	[0.22, 0.81]	[0.21, 0.86]	[1.65, 3.81]	[0.36, 0.98]	[0.62, 1.07]	[0.02, 8.46]	[1.75, 25.30]
		*p* value	***0.01***	***0.02***	***<0.0001***	***0.04***	0.14	0.53	***0.005***
		I^2^	83%	86%	23%	66%	0%	n/a	0%
HAIC methods subgroup analysis	With port system^c^	HR/RR	0.36	0.09	2.64	0.41	1.18	0.36	3.26
		95%CI	[0.17, 0.79]	[0.03, 0.27]	[1.44, 4.85]	[0.16, 1.07]	[0.72, 1.94]	[0.02, 8.46]	[0.14, 76.10]
		*p* value	***0.01***	***<0.0001***	***0.002***	0.07	0.52	0.53	0.46
		I^2^	67%	n/a	0%	59%	0%	n/a	n/a
	Without port system^d^	HR/RR	0.60	0.61	3.03	0.55	0.71	0.63	2.68
		95%CI	[0.50, 0.72]	[0.52, 0.72]	[2.11, 4.35]	[0.40, 0.76]	[0.56, 0.90]	[0.12, 3.21]	[1.41, 5.08]
		*p* value	***<0.00001***	***<0.00001***	***<0.00001***	***0.0003***	***0.006***	0.57	***0.003***
		I^2^	0%	5%	47%	53%	43%	0%	24%
HAIC regimen subgroup analysis	Using FOLFOX-HAIC^e^	HR/RR	0.48	0.51	2.67	0.50	0.82	0.56	2.37
		95%CI	[0.32, 0.72]	[0.36, 0.73]	[2.00, 3.57]	[0.34, 0.74]	[0.68, 0.98]	[0.13, 2.38]	[1.54, 3.66]
		*p* value	**0.0004**	**0.0002**	***<0.00001***	**0.0005**	**0.03**	0.43	**<0.0001**
		I^2^	75%	72%	0%	0%	0%	0%	17%
	Using cisplatin + fluorouracil combined HAIC^f^	HR/RR	0.53	n/a	3.09	0.57	1.14	n/a	n/a
		95%CI	[0.30, 0.92]	n/a	[0.66, 14.35]	[0.25, 1.31]	[0.63, 2.06]	n/a	n/a
		*p* value	**0.03**	n/a	0.15	0.19	0.66	n/a	n/a
		I^2^	0%	n/a	34%	40%	0%	n/a	n/a

a , Reference (Li et al., 2022; He et al., 2017)

b Reference (An et al., 2021; Hu et al., 2020; Li et al., 2021)

c References (Sumie et al., 2003; Kim et al., 2010; Hu et al., 2020)

d References (He et al., 2017; An et al., 2021; Li et al., 2021; Chen et al., 2022; Li et al., 2022)

e References (He et al., 2017; Hu et al., 2020; An et al., 2021; Li et al., 2021; Li et al., 2022)

f References (Sumie et al., 2003; Kim et al., 2010).

BCLC, barcelona clinic liver cancer; HR, hazard ratio; RR, risk ratio; OS, overall survival; PFS, progression-free survival; PR, partial response; PD, progressive disease; SD, stable disease.

To highlight the results with statistical significance.

In order to reduce the impact of treatment selection bias in analysis, we further conducted subgroup analysis among studies with propensity score matching data (He et al., 2017; Li et al., 2021; Chen et al., 2022; Li et al., 2022; He et al., 2017; Li et al., 2021; Chen et al., 2022; Li et al., 2022; He et al., 2017; Li et al., 2021; Chen et al., 2022; Li et al., 2022). Pooled results after adjustment of tumour background showed that in both BCLC stage A-B and stage C, the HAIC group had better OS (HR = 0.58, 95%CI [0.43, 0.78]; HR = 0.59, 95%CI [0.40, 0.87]), PFS(HR = 0.57, 95%CI [0.45, 0.72]; HR = 0.66, 95%CI [0.49, 0.89]) and PR (RR = 2.56, 95%CI [1.76, 3.72]; RR = 3.51, 95%CI [2.16, 5.69]) as well as higher subsequent resection rate (RR = 1.96, 95%CI [1.18, 3.25]; RR = 7.78, 95%CI [1.78, 33.99]) compared to the TACE group, but with tumour progression (stage A-B→C) the priority of HAIC on reducing PD, ALT level and hyperbilirubinemia decreased (Supplementary Table S5).

### GRADE summary of findings and TSA results

The certainty of evidence after GRADE assessment showed that though most findings were with low/very low quality, outcome of PR presented high quality of evidence level: per 1,000 patients received treatment, 280 more in the HAIC group would have PR compared to the TACE group (Table 4). This finding was further tested using TSA. It is clear that the cumulative z-curve crossed the upper trial sequential monitoring boundary after adding data from the study by (Chen et al., 2022) indicating that HAIC is effective in increasing PR compared to TACE where α = 0.05, a power of 80% and RRR of 25% were set. Although the total number of subjects (*n* = 1,060) did not reach the required size of 3,648, a stable conclusion could already be drawn from the current data (Supplementary Figure S5).

**TABLE 4:** Grade summary of finding table.

Outcomes	№ of participants (studies)follow-up	Certainty of the evidence (GRADE)	Relative effect (95% CI)	Anticipated absolute effects	
				Risk with [TACE]	Risk difference with [HAIC]
Overall Survival (OS) assessed with: HR	981 (7 non-randomised studies)	⊕⊕○○ Low^a^ ^,^ ^b^ ^,^ ^c^	**HR 0.53** (0.40–0.69) [Overall Survival]	Low	
				135 per 1,000	**61 fewer per 1,000** (79 fewer to 40 fewer)
Progression-free survival (PFS) assessed with: HR	956 (6 non-randomised studies)	⊕⊕○○ Low^a^ ^,^ ^b^ ^,^ ^c^	**HR 0.54** (0.40–0.72) [progression-free survival]	Low	
				50 per 1,000	**23 fewer per 1,000** (30 fewer to 14 fewer)
Partial Response (PR) assessed with: RR	1,060 (8 observational studies)	⊕⊕⊕⊕ High^a^ ^,^ ^d^	**RR 2.87** (2.18–3.78)	150 per 1,000	**280 more per 1,000** (177 more to 416 more)
Stable Disease (SD) assessed with: RR	1,060 (7 observational studies)	⊕○○○ Very low^a^ ^,^ ^d^	**RR 0.77** (0.62–0.96)	416 per 1,000	**96 fewer per 1,000** (158 fewer to 17 fewer)
Progressive Disease (PD) assessed with: RR	1,060 (8 observational studies)	⊕○○○ Very low^a^ ^,^ ^b^ ^,^ ^d^ ^,^ ^e^	**RR 0.54** (0.41–0.72)	373 per 1,000	**171 fewer per 1,000** (220 fewer to 112 fewer)
Intervention related Mortality assessed with: RR	1,060 (8 observational studies)	⊕○○○ Very low^a^ ^,^ ^d^	**RR 0.56** (0.13–2.38)	9 per 1,000	**4 fewer per 1,000** (8 fewer to 13 more)
Resection assessed with: RR	796 (5 observational studies)	⊕⊕○○ Low^a^ ^,^ ^d^	**RR 2.37** (1.54–3.66)	60 per 1,000	**104 more per 1,000** (33 more to 161 more)

The risk in the intervention group (and its 95% confidence interval) is based on the assumed risk in the comparison group and the relative effect of the intervention (and its 95% CI). CI, confidence interval; HR: hazard Ratio; RR: risk ratio. GRADE, Working Group grades of evidence. High certainty: we are very confident that the true effect lies close to that of the estimate of the effect. Moderate certainty: we are moderately confident in the effect estimate: the true effect is likely to be close to the estimate of the effect, but there is a possibility that it is substantially different. Low certainty: our confidence in the effect estimate is limited: the true effect may be substantially different from the estimate of the effect. Very low certainty: we have very little confidence in the effect estimate: the true effect is likely to be substantially different from the estimate of effect.

a Most studies included are non-randomized, there exits unavoidable selection bias.

b Significantly statistical heterogeneity.

c Basic study design was define as non-randomised as only one study is RCT, whereas others were all observational cohort studies. But due to the existence of RCT, the real overall certainty of evidence maybe even higher.

d Basic study design was define as observational as only one study is RCT, whereas others were all observational cohort studies. But due to the existence of RCT, the real overall certainty of evidence maybe even higher.

e Clear asymmetry observed from publication bias funnel plot.

To highlight the results with statistical significance.

## Discussion

Compared with conventional chemotherapy, transarterial chemotherapies such as HAIC and TACE appear to provide higher drug concentration in liver tumours and effectively reduce systemic side effects (Ikeda et al., 2018; Sigurdson et al., 1987). In addition, through constant infusion by a fixed-microcatheter, theoretically the high concentration persists for longer in HAIC compared to TACE (Gao et al., 2018). Though long-time infusion (usually >24 h) and temporarily placed-catheter increased the burden of clinical care compared to the same-day discharge after TACE, from a health economics perspective the improved anti-tumour effect of HAIC was worthwhile. Our study showed that patients with unresectable HCCs who received HAIC as initial therapy had longer OS, PFS and better PR than those who received TACE. Furthermore, prolonged chemotherapy infusion seemed to be associated with well tolerated liver toxicity. Liver decompensation or procedure-related death rarely occurred in the HAIC group. This therapy seemed to be relatively safe in those patients with predominately hepatitis B (916/1,060, 86.42%) related HCC. Of note, the strict patient inclusion criteria maybe the key in achieving the high safety: patients included in this meta-analysis were predominately Child-Pugh Grade A with only a small number of Grade B (Score 7) (Supplementary Table S3).

A significant difference from HAIC is that TACE also embolizes the feeding tumour artery resulting in necrosis. Intra-tumoral necrosis may weaken the adhesive potential of the tumour thus facilitate release of cancer cells from the tumour site with dislodgement into the bloodstream (Adachi et al., 1993; Ravaioli et al., 2004; Ha et al., 2016). Moreover, large HCCs usually develop an abundant collateral circulation which is difficult to completely embolize while the transient hypoxic microenvironment caused by embolism could induce the upregulation of hypoxia-inducible factors (HIFs) which in turn promote tumour progression (Muz et al., 2015; Al Tameemi et al., 2019). Similar findings were observed in this study, with more patients experiencing PD after TACE in contrast to those who received HAIC. The higher PD rate may also explain why the TACE group had lower OS and PFS. In addition, the arterial embolism could cause adverse events including ectopic embolism, severe pain and liver decompensation, especially in those with PVTT (Garwood et al., 2013; Blackburn and West, 2016; Tan et al., 2019; Pachev et al., 2021). It is consistent with our results that the risk of Grade III-IV fevers, hyperbilirubinemia and abnormal liver function (elevated ALT level) were higher in patients receiving TACE.

TACE has been a long-standing first line locoregional therapy for intermediate stage HCC and is recommended by many national and international guidelines. Efforts to extend the application of TACE in advanced HCC have also been made over past decades. However, with renewed interest in the use of HAIC, benefits of this therapy in treating both intermediate and advanced stage HCC are being reported. To those patients, there is controversy over whether the use of HAIC should be considered as a treatment option in place of TACE. Sorafenib is a multi-target and multi-kinase inhibitor for the treatment of liver cancer and has been the first line treatment for advanced HCC^10^. A RCT conducted in Korea showed that the ORR of advanced HCC with PVTT was higher in the HAIC group than that in the sorafenib group. Both OS and time-to-progression (TTP) were longer in patients receiving HAIC treatment (14.9 vs. 7.2 months, *p* = 0.012 and 4.4 vs. 2.7 months, *p* = 0.010) (Choi et al., 2018). A phase III trial (FOHAIC-1) reported by Lyu et al. showed that HAIC was associated with improved prognosis in advanced HCC even patients were with a high intrahepatic disease burden compared to the sorafenib group (Lyu et al., 2022). Kodama et al. also found that HAIC was significantly better than sorafenib as primary treatment in microvascular invasion (MVI) and non-TACE refractory cases (Kodama et al., 2018).

In addition, with the availability of new combinations of chemotherapy agents such as FOLFOX (fluorouracil/leucovorin/oxaliplatin), the potential application of HAIC in the treatment of advanced HCC has attracted more attention in recent years. A phase II trial validated the use of FOLFOX in HAIC with favourable 6- and 12-month survival rates and a higher response rate with significant improvement in patients’ quality of life after treatment (*p* < 0.001) (Lyu et al., 2018). In this study, patients who received FOLFOX-HAIC had better OS, PFS, PR and PD as well as higher subsequent resection rate in both BCLC stage A-B and stage C HCC compared to those who received TACE as initial treatment. Compared with traditional cisplatin plus fluorouracil regimen, the efficacy of FOLFOX-HAIC appears to be better (Table 3).

High response rates achieved after TACE makes subsequent liver resection possible in selected cases with naturally unresectable HCCs (Shi et al., 2012; Allard et al., 2015; Kang et al., 2017). This meta-analysis showed that compared to TACE, HAIC had a higher PR rate making it potentially attractive as transformational therapy for unresectable HCC. GRADE assessment and TSA test proved that this finding has both high certainty and stability. The higher the rates of CR or PR, the more likely it is to achieve clinically significant tumour downstaging (Zhang et al., 2016). Pooled RRs in our study further confirmed this possibility: the resection rate of patients in the HAIC group was significantly higher than that in the TACE group, HAIC shows potential in prolonging life with better quality. HCC have rapid disease progression and intermediate stage HCC can progress to advanced stage limiting potential treatment options. After adjusting for tumour burden with propensity score matching data, we found that for patients with BCLC stage A-B HCC HAIC could better lower the PD rate than TACE, whereas for stage C, there was no difference between the two groups. Therefore, for intermediate stage HCC, HAIC should be considered as an alternative option for treatment.

Only two cases of CR (2/1060, 0.19%) were observed in this analysis which suggests that monotherapy may not be optimal treatment. Combining HAIC with other therapies may provide better therapeutic effects for advanced HCC. A small number of studies from Asia have shown that the ORR, OS and PFS of sorafenib combined with HAIC are significantly better than sorafenib monotherapy (Kondo et al., 2019; He et al., 2019). Mei et al. (2021) reported that HAIC combined with PD-1 inhibitors plus Lenvatinib was associated with a better treatment response and survival in patients with advanced HCC compared to PD-1 inhibitors plus Lenvatinib alone. For HCC with macrovascular invasion, radical resection was performed after concurrent chemoradiation therapy (CCRT) combined with HAIC with a conversion rate of 26.5% (26/98) (Chong et al., 2018). Currently using TACE in patients with PVTT remains controversial while HAIC could be used to complement other modalities of treatments. Han et al. (2016) reported eight patients with locally advanced HCC and PVTT who received CCRT followed by HAIC and underwent living donor liver transplant. Median overall survival time from initial diagnosis reached 33 months. Kosaka et al. (2021) also reported that the combination of HAIC and three-dimensional conformal radiation therapy for advanced HCC with tumour thrombosis of the main trunk or bifurcation of the portal vein should be considered as a good option in selected patients. The ORR was 13.7% in main tumour and 51% in the PVTT. (Hu et al., 2020) reported that HAIC might be a much better option than TACE for patients with major PVTT, similar findings were also observed in this study in patients with Vp3-Vp4 PVTT (Supplementary Table S4).

The strengths of this study included the use of a pre-registered protocol, performing a comprehensive literature search and independent study screening/data extraction/quality assessment, conducting deep subgroup analysis based on different tumour characters, pooling propensity score matching data to adjust tumour background and performing GRADE assessment for the certainty of evidence and TSA for findings with high certainty. However, the small number of studies (*n* = 8) involved may limit the calculation power of our analysis. These studies have mainly been conducted in Asia (South Korea/Japan/China) and predominately in patients with hepatitis B related HCC. The clinical application of HAIC in Europe and America is limited with no relevant data available. In addition, the definition of “unresectable HCC” and the criteria for surgical resection post-HAIC or post-TACE may vary among different centres. These limitations suggest that multi-centre, large scale prospective or randomised studies are still needed to see whether the results from predominately hepatitis B patients can be replicated in other liver disease background and in a Western context.

In conclusion, HAIC presents advantages over TACE in terms of prolonging survival, fewer severe adverse event, better treatment response and tumour downstaging in unresectable HCC. These encouraging results may update the treatment algorithm for HCC though validation in other liver disease background and Western cohort is warranted.

## Data Availability

The original contributions presented in the study are included in the article/Supplementary Material, further inquiries can be directed to the corresponding authors.
