# Supplementary figures and images for: Hepatic arterial infusion chemotherapy versus transarterial chemoembolization for unresectable hepatocellular carcinoma: A systematic review with meta-analysis
Source: Front Bioeng Biotechnol. 2022 Sep 27;10:1010824. doi: 10.3389/fbioe.2022.1010824 (PMC9551027; doi:10.3389/fbioe.2022.1010824)

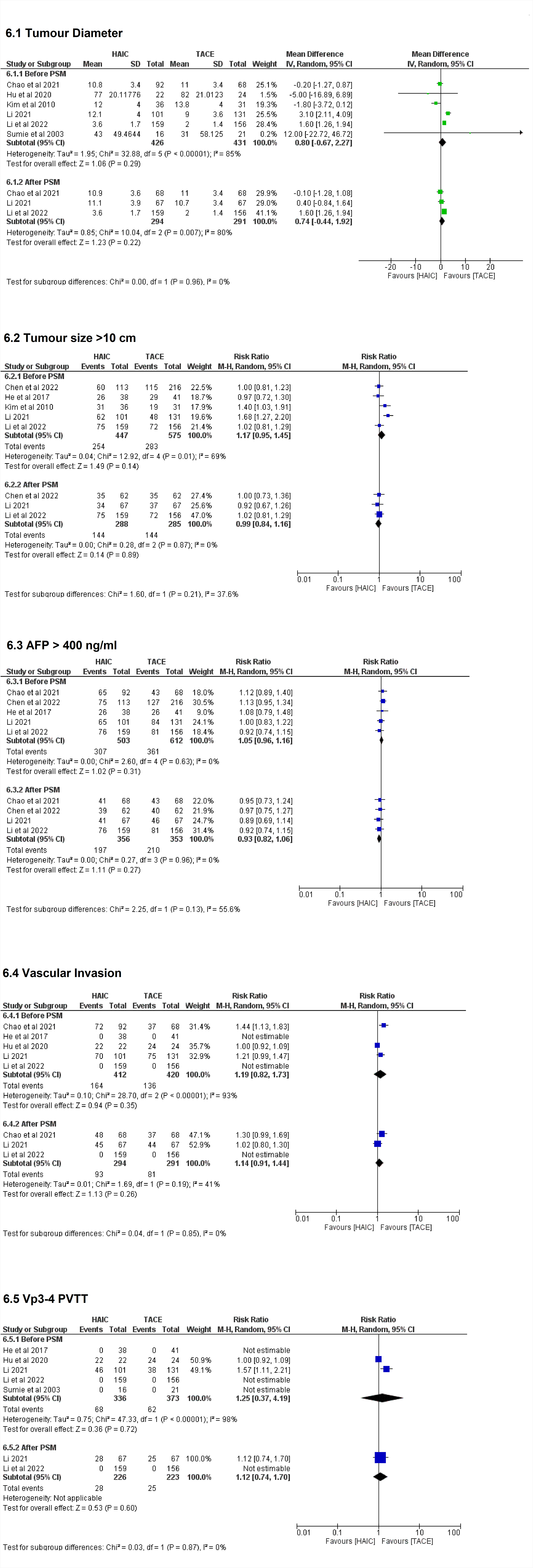

Supplement: Supplementary file 1 [file Image6.TIF]

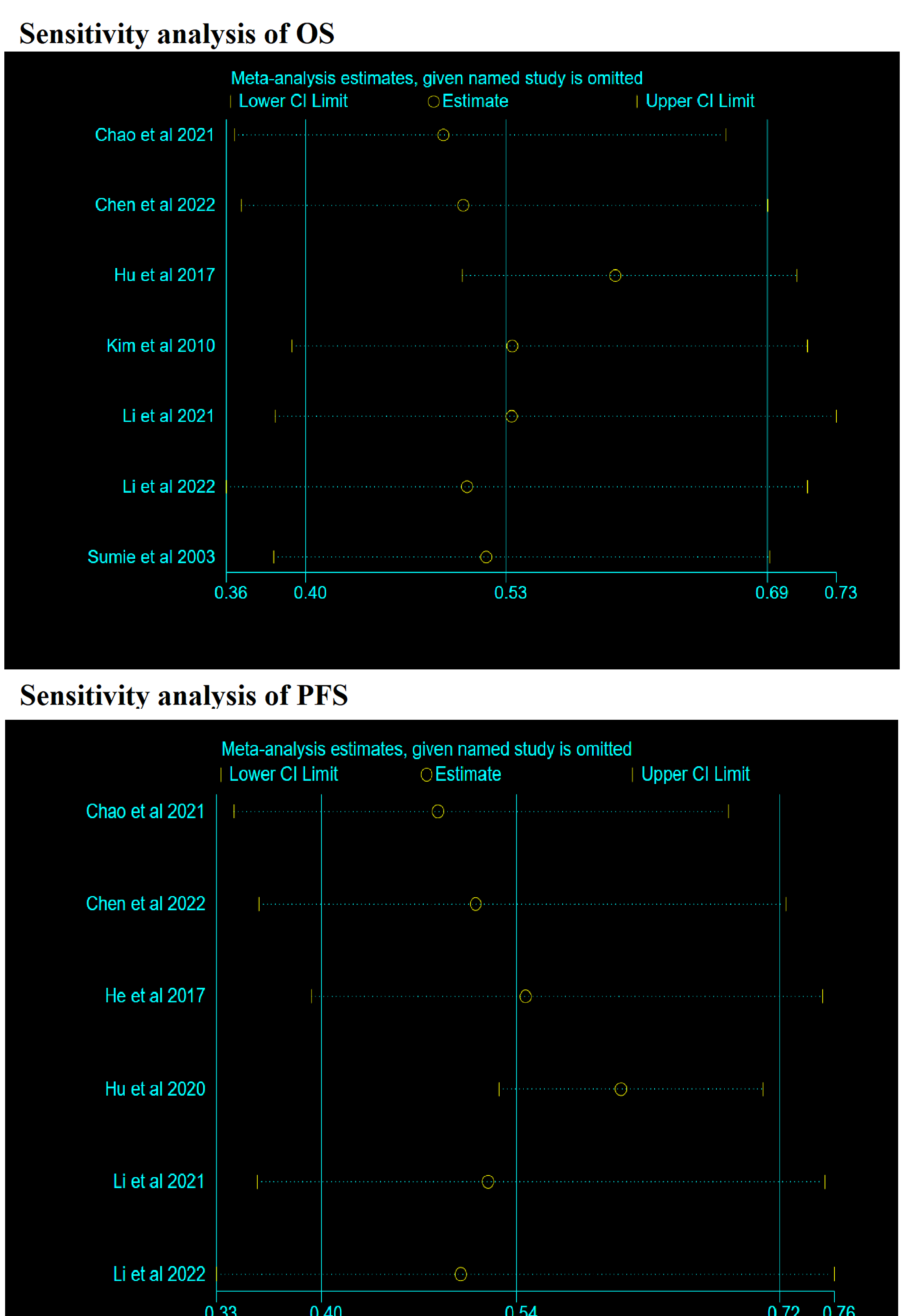

Supplement: Supplementary file 3 [file Image4.TIF]

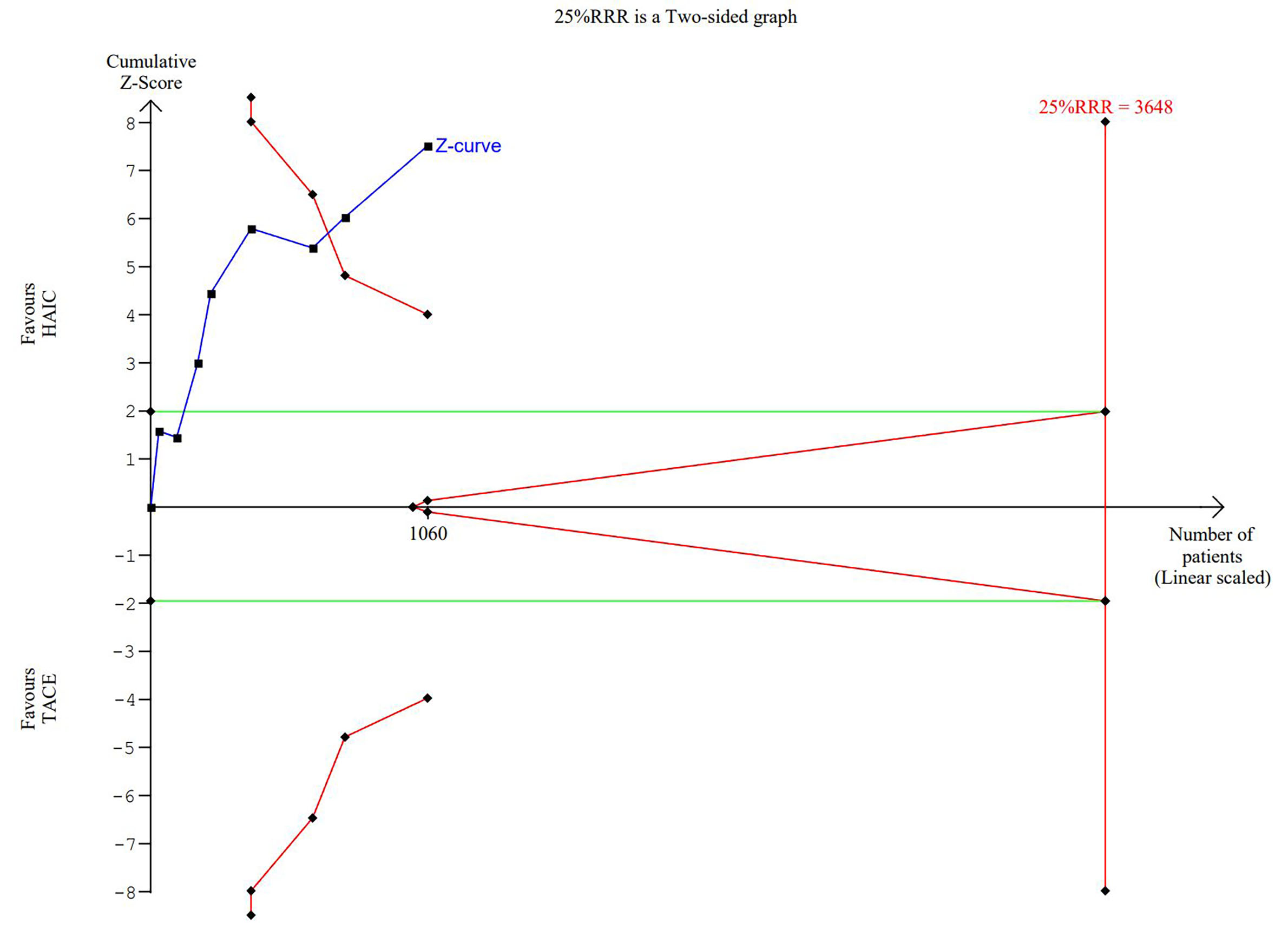

Supplement: Supplementary file 5 [file Image5.JPEG]

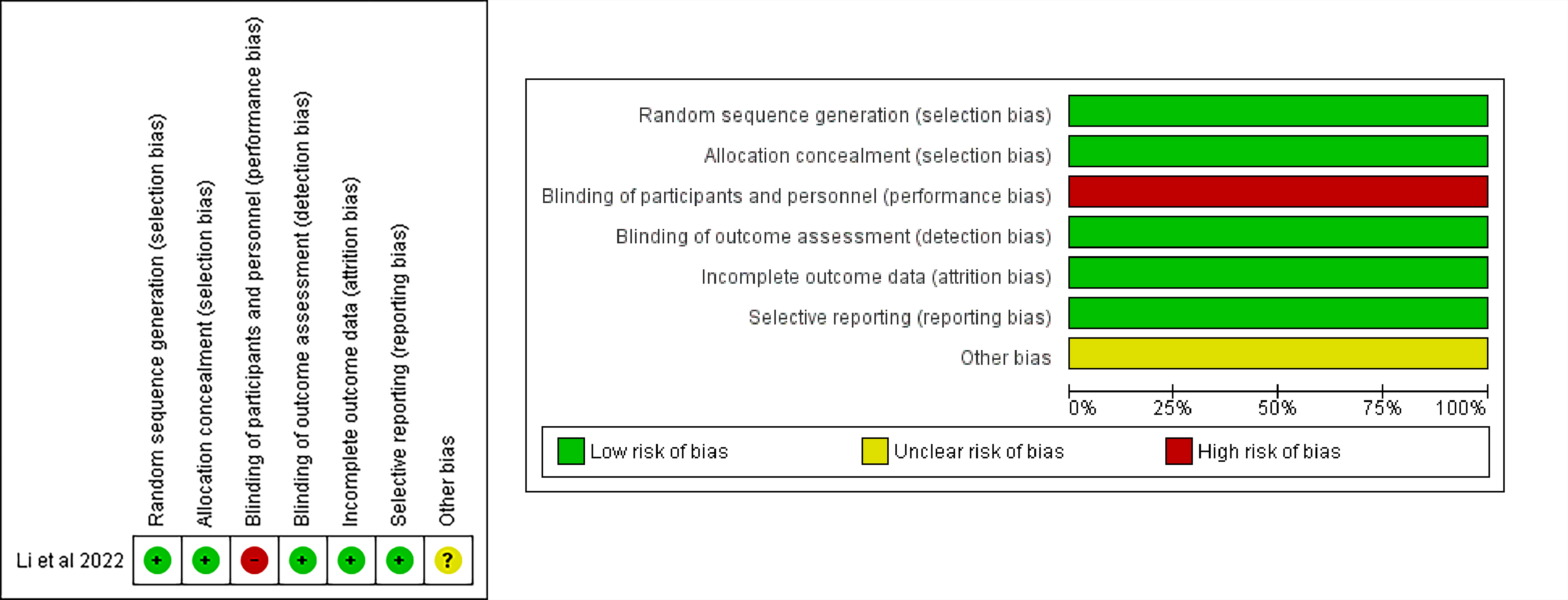

Supplement: Supplementary file 6 [file Image1.TIF]
