# Supplementary material for: Hepatic arterial infusion chemotherapy versus transarterial chemoembolization for unresectable hepatocellular carcinoma: A systematic review with meta-analysis
Source: Front Bioeng Biotechnol. 2022 Sep 27;10:1010824. doi: 10.3389/fbioe.2022.1010824 (PMC9551027; doi:10.3389/fbioe.2022.1010824)
Supplement: Supplementary file 7 [file DataSheet1.docx]

Supplementary Table 1 Search Strategies

| ***Name of database*** | ***Time span*** | ***Search strategy*** | ***Items*** |
| --- | --- | --- | --- |
| The Cochrane Central Register of Controlled Trials (CENTRAL) in The Cochrane Library | Issue 6，2021 | **#1** ((hepatocellular or liver cell or hepatic) and (carcinoma* or tumo* or cancer)) or HCC or hepatoma*:ti,ab,kw (Word variations have been searched)  **#2** ((liver or hepatic or Intra) and (infusion)) or HAIC or HAI: ti,ab,kw (Word variations have been searched)  **#3** ((transcatheter or transarterial) and (emboli* or chemoemboli*)) or TAE or TACE:ti,ab,kw (Word variations have been searched)  **#4** MeSH descriptor: [Carcinoma, Hepatocellular] explode all trees  **#5** MeSH descriptor: [arterial infusion] explode all trees  **#6** MeSH descriptor: [Embolization, Therapeutic] explode all trees  **#7 #1** or **#4**  **#8 #2** or **#5**  **#9 #3** or **#6**  **#10 #7** and **#8** and **#9** | n=15 |
| PubMed | 1950- July 2022 | ((((((((((carcinoma, hepatocellular[MeSH Terms]) OR hepatocellular carcinoma[Title/Abstract]) OR HCC[Title/Abstract]) OR hepatocarcinoma[Title/Abstract]) OR hepatomas[Title/Abstract]) OR liver carcinoma[Title/Abstract]) OR liver cancer[Title/Abstract]) OR liver cell carcinoma[Title/Abstract])) AND ((((hepatic arterial infusion chemotherapy[Title/Abstract]) OR hepatic arterial infusion[Title/Abstract]) OR HAIC[Title/Abstract]) OR HAI[Title/Abstract])) AND ((((Embolization, Therapeutic[MeSH Terms])OR((transcatheter or trans arterial) and (emboli* or chemoemboli*)) OR TAE[Title/Abstract]) or TACE[Title/Abstract])) | n=173 |
| EMBASE | 1966- July 2022 | (((hepatocellular AND ('carcinoma'/exp OR carcinoma) OR hcc OR 'hepatocarcinoma'/exp OR hepatocarcinoma OR hepatomas OR 'liver'/exp OR liver) AND ('carcinoma'/exp OR carcinoma) OR 'liver'/exp OR liver) AND ('cancer'/exp OR cancer) OR 'liver'/exp OR liver) AND ('cell'/exp OR cell) AND ('carcinoma'/exp OR carcinoma) AND ((hepatic AND arterial AND ('infusion'/exp OR infusion) AND ('chemotherapy'/exp OR chemotherapy) OR hepatic) AND arterial AND ('infusion'/exp OR infusion) OR haic OR hai) AND ((transcatheter OR transarterial )AND (emboli* OR chemoemboli*) OR tae OR tace) AND [abstracts]/lim AND [embase]/lim and [english]/lim | n=196 |
| Web of Science | 1945- July 2022 | ((Topic: ((transcatheter OR transarterial )AND (emboli* OR chemoemboli*) OR tae OR tace) AND Topic: ((((((hepatocellular carcinoma OR HCC) OR hepatocarcinoma) OR hepatomas) OR liver carcinoma) OR liver cancer) OR liver cell carcinoma)) AND Topic: (((hepatic arterial infusion chemotherapy OR hepatic arterial infusion) OR HAIC) OR HAI)) Refined by: LANGUAGES: ( ENGLISH ) | n=144 |

Supplementary Table 2 Critical appraisal using the Newcastle-Ottawa Quality Assessment Scale for Cohort Study

|  | Selection | | | | Comparability | Outcome | | | Total |  |
| --- | --- | --- | --- | --- | --- | --- | --- | --- | --- | --- |
| Resource | Representativeness  of the exposed cohorts | Selection of the  non-exposed cohorts | Ascertainment of exposure | Demonstration that outcome of interest |  | Ascertainment of outcome | Length of follow-up | Adequacy of follow-up |  |  |
| He at al 2017 | ★ | ★ | ★ | ★ | ★★ | ★ | ★ | - | ★★★★★★★ | |
| Hu et al 2020 | ★ | ★ | ★ | _ | ★ | ★ | ★ | ★ | ★★★★★★★ | |
| Kim et al 2010 | ★ | ★ | ★ | _ | ★ | ★ | _ | _ | ★★★★★ | |
| Sumie et al 2003 | ★ | ★ | ★ | ★ | ★ | - | ★ | _ | ★★★★★★ | |
| Li et al 2021 | ★ | ★ | ★ | ★ | ★★ | ★ | ★ | ★ | ★★★★★★★★★ | |
| Chao et al 2021 | ★ | ★ | ★ | ★ | ★★ | ★ | ★ | - | ★★★★★★★★ | |
| Chen et al 2022 | ★ | ★ | ★ | ★ | ★★ | ★ | - | - | ★★★★★★★ | |

Supplementary Table 3 Patient selection criteria of included studies for HCC patients.

| Author | Year | Included patients |  | Excluded patients |  |
| --- | --- | --- | --- | --- | --- |
| Li et al | 2022 | 1. age 18 years or older 2. with unresectable Barcelona clinic liver cancer A-B HCC 3. the largest lesion ≥7 cm per RECIST version 1.1 4. Child-Pugh A class liver function 5. Eastern Cooperative Oncology Group performance status (ECOG PS) of 0-1, 6. no previous treatment for HCC 7. WBC count ≥ 3.0x10^9^ per L, absolute neutrophils ≥1.5x 10^9^ per L, platelet cell count ≥ 75x10^9^ per L, AST   and ALT ≤5 times the upper limit of the normal range,  creatinine clearance rate of ≥ 1.5 times the upper limit of  the normal range, and left ventricular ejection ≥ 45% | | 1. hepatic decompensation (a history of esophageal or gastric variceal bleeding or hepatic encephalopathy) 2. pregnancy or breastfeeding 3. with other invasive malignant diseases |  |
| Chen et al | 2022 | 1. HCC with ≥ 4 tumours or portal vein tumour thrombosis (China Liver Cancer Stage IIb–IIIa) 2. age≥18 years 3. Eastern Cooperative Oncology Group (ECOG) 4. performance status of 0–2 5. Child–Pugh class A or B   appropriate hematologic values (leukocyte count>3000/mm3, platelet count>50,000/mm3) | | 1. extrahepatic spread 2. treatment was combined with other therapies |  |
| Chao et al | 2021 | 1. age 18–75 years 2. Eastern Cooperative Oncology Group (ECOG) performance status < 2 3. Child-Pugh class A liver function 4. With advanced HCC confirmed by EASL and AASLD guidelines 5. Patients refused first-line multi-targeted tyrosine kinase inhibitors (TKIs) treatment | | 1. patients underwent any treatment before IAT 2. history of any systemic therapy 3. HCC combined with other malignancies 4. Child-Pugh class B or C liver function 5. clinical and imaging data missing 6. lost to follow-up > 6 months | |
| Li et al | 2021 | 1. age between 18-75 years 2. confirmed diagnosis of advanced HCC based on BCLC staging without prior locoregional or systemic treatment 3. adequate function of blood/bone marrow (leukopenia count >3.0×109/L, haemoglobin >8.0 g/L, and platelet count >60×109/L), liver (ALT and AST <5 times the upper limit of the normal range, albumin >2.8 g/L, total bilirubin <2.8 g/L), renal (serum creatinine <1.5 times the upper limit of the normal range) 4. and coagulation (prothrombin time <6 s) function 5. controlled arterial hypertension | | 1. prior treatment for HCC 2. prior treatment with oxaliplatin or fluorouracil previous liver surgery 3. extensive liver cancer metastasis 4. the sum of the size of the largest EHS lesions [in cm] and the number of EHS lesions exceeding 5. no baseline imaging assessment or lost to follow-up |  |
| Hu et al | 2020 | 1. with major portal vein tumour thrombosis 2. ECOG performance status≤2 3. Child–Pugh classification of either A or B7 4. at least 1 measurable lesion according to the RECIST version 1.1 5. absence of severe ascites 6. white blood cell count ≥3.5x10^9^/L or an absolute neutrophil count ≥ 1.5x 10^9^/L; platelet count>75 x10^9^/L; INR ≤1.5; AST and ALT ≤5 X upper limit of the normal; serum creatinine < 2.0 mg/dL; creatinine clearance rate of ≤1.5 X upper limit of the normal; and left ventricular ejection ≥45% |  | 1. extrahepatic primary malignancy 2. intractable comorbid medical illness. 3. Combined therapy of HAIC and TACE 4. Received other local treatment during the arterial-directed therapy period 5. Incomplete or lacking follow-up information |  |
|  |  |  |  |  |  |
| He et al | 2017 | 1. the sum of diameters of all lesions longer than 10 cm with the maximum lesion longer than 7 cm. 2. age between 18 and 75 years. 3. the tumour was not amenable to surgical resection or any other curative treatment. 4. platelet count ≥ 75,000/μL, haemoglobin ≥ 8.5 g/dL, total bilirubin ≤ 30 mmol/L, and serum albumin ≥ 32 g/L. 5. the absence of cirrhosis or a cirrhotic status of Child–Pugh class A only |  | 1. a previous history of treatment for HCC. 2. vascular invasion or distant metastasis. 3. severe underlying cardiac or renal diseases. 4. a second primary malignancy. 5. Child Pugh C or ECOG score >2 |  |
|  |  |  |  |  |  |
| Kim et al | 2010 | 1. with main portal invasion 2. diffuse bilobar involvement and/or refractory to surgical resection or nonsurgical intervention 3. age between 18 and 70 years 4. Child-Pugh score of 5 or 6 (Grade A) 5. ECOG performance status <2, 6. serum creatinine level ≤1.5 mg/dL, aminotransferase < 200 IU/mL, absolute neutrophil count ≥1,500/mm3, platelet count ≥75,000/mm3 and haemoglobin ≥ 10 g/dL |  | 1. extrahepatic primary malignancy 2. metastasis and/or intractable comorbid medical illness. |  |
| Sumie et al | 2003 | 1. advanced stage of HCC as assessed by large size, multiplicity, or vascular involvement 2. age between 18 and 80 years 3. Child-Pugh (A-B) 4. TNM stage II-IV |  | n/a |  |

n/a, not available. ALT, alanine transaminase; AST, aspartate transaminase; ECOG, Eastern Cooperative Oncology Group; HAIC, hepatic arterial infusion chemotherapy; HCC, hepatocellular carcinoma; INR, international normalized ratio; RECIST, Response Evaluation Criteria in Solid Tumours; TACE, transarterial chemoembolization

Supplementary Table 4 Subgroup analysis of all included studies on PFS, OS and PD

| **Outcomes** | **Subgroup** | **Studies** | **HR and 95%CI** | **Test for overall effect** | **Heterogeneity** |
| --- | --- | --- | --- | --- | --- |
| **PFS** | All patients with Vp3 or Vp4 PVTT | 1 | HR=0.09, 95%CI [0.03, 0.27] | *P<0.0001* | *n/a* |
|  | A portion of patients with Vp3-Vp4 PVTT | 2 | HR=0.66, 95%CI [0.49, 0.89] | *P=0.006* | *P=0.20, I^2^ =40%* |
|  | No patients with Vp3-Vp4 PVTT | 2 | HR=0.56, 95%CI [0.45, 0.70] | *P<0.00001* | *P=0.56, I^2^ =0* |
|  |  |  |  |  |  |
| **OS** | All patients with Vp3 or Vp4 PVTT | 1 | HR=0.17, 95%CI [0.08, 0.36] | *P<0.00001* | *n/a* |
|  | A portion of patients with Vp3-Vp4 PVTT | 3 | HR=0.57, 95%CI [0.43, 0.77] | *P<0.00001* | *P=0.29, I^2^ =20%* |
|  | No patients with Vp3-Vp4 PVTT | 2 | HR=0.59, 95%CI [0.44, 0.78] | *P=0.0002* | *P=0.83, I^2^ =0* |
| **n/a:** not available; **CI:** confidence interval**; HR:** Hazard Ratio; **OS:** overall survival; **PD**: progressive disease; **PVTT:** portal vein tumour thrombosis; **PFS:** progression free survival; **Vp3:** presence of portal vein tumour thrombosis in the first-order branches of the portal vein, **Vp4:** presence of portal vein tumour thrombosis in the main trunk of the portal vein or a portal vein branch contralateral to the primary involved lobe (or both) | | | | | |

Supplementary Table 5. Comparisons of outcomes and adverse events after propensity score matching

|  | **Patients with BCLC stage A-B HCC ^*^** | | |  | **Patients with BCLC stage C HCC ^**^** | | |
| --- | --- | --- | --- | --- | --- | --- | --- |
|  | HR/RR | 95%CI | P value |  | HR/RR | 95%CI | P value |
| **OS** | 0.58 | [0.43, 0.78] | **0.0004** |  | 0.59 | [0.40, 0.87] | **0.007** |
| **PFS** | 0.57 | [0.45, 0.72] | **<0.00001** |  | 0.66 | [0.49, 0.89] | **0.006** |
| **Treatment Response** |  |  |  |  |  |  |  |
| ***PR*** | 2.56 | [1.76, 3.72] | **<0.00001** |  | 3.51 | [2.16, 5.69] | **<0.00001** |
| ***PD*** | 0.40 | [0.25, 0.64] | **0.0001** |  | 0.57 | [0.30, 1.08] | 0.09 |
| ***SD*** | 0.85 | [0.65, 1.12] | 0.24 |  | 0.74 | [0.39, 1.42] | 0.37 |
| **Mortality** | 0.98 | [0.14, 6.88] | 0.98 |  | n/a | n/a | n/a |
| **Resection** | 1.96 | [1.18, 3.25] | **0.009** |  | 7.78 | [1.78, 33.99] | **0.006** |
| **Adverse Events** |  |  |  |  |  |  |  |
|  |  |  |  |  |  |  |  |
| ***Fever*** | 0.14 | [0.01, 2.69] | 0.19 |  | 0.43 | [0.02, 10.48] | 0.61 |
| ***Leukopenia*** | 3.92 | [0.44, 34.72] | 0.22 |  | 2.61 | [0.03, 259.97] | 0.68 |
| ***Diarrhea*** | 6.87 | [0.36, 131.89] | 0.20 |  | n/a | n/a | n/a |
| ***Elevated ALT level*** | 0.43 | [0.23, 0.78] | **0.006** |  | 0.14 | [0.01, 1.45] | 0.10 |
| ***Hyperbilirubinemia*** | 0.11 | [0.01, 0.85] | **0.03** |  | n/a | n/a | n/a |
| ***Neutropenia*** | 3.92 | [0.44, 34.72] | 0.22 |  | 1.58 | [0.07, 37.74] | 0.78 |
| ***Anaemia*** | 2.94 | [0.12, 71.72] | 0.51 |  | n/a | n/a | n/a |
| ***Thrombocytopenia*** | 1.96 | [0.50, 7.71] | 0.33 |  | 2.59 | [0.48, 13.88] | 0.27 |

* N=315 (HAIC, n=159; TACE, n=156); ** N= 270 (HAIC, n=135; TACE, n=135)

BCLC, Barcelona Clinic Liver Cancer; HR, hazard ratio; RR, risk ratio; OS, overall survival; PFS, progression-free survival; PR, partial response; PD, progressive disease; SD, stable disease
